# Molecular Detection and Phylogenetic Characterization of *Bartonella bovis* in Dairy Cattle From Central Thailand

**DOI:** 10.1155/tbed/3831037

**Published:** 2026-08-06

**Authors:** Wittawat Wechtaisong, Kritsada Thongmeesee, Chalida Sri-in, Aung Aung, Wisa Tiyamanee, Satoru Konnai, Sonthaya Tiawsirisup

**Affiliations:** ^1^ Faculty of Medicine, Vongchavalitkul University, Nakhon Ratchasima 30000, Thailand, vu.ac.th; ^2^ Center of Excellence in Animal Vector-Borne Diseases, Veterinary Parasitology Unit, Department of Veterinary Pathology, Faculty of Veterinary Science, Chulalongkorn University, Bangkok 10330, Thailand, chula.ac.th; ^3^ Department of Veterinary Science, Faculty of Veterinary Science, Prince of Songkla University, Songkhla 90110, Thailand, psu.ac.th; ^4^ Department of Disease Control, Faculty of Veterinary Medicine, Hokkaido University, Sapporo 060-0818, Japan, hokudai.ac.jp; ^5^ Institute for Vaccine Research and Development (HU-IVReD), Hokkaido University, Sapporo 001-0021, Japan, hokudai.ac.jp

**Keywords:** bartonellosis, livestock health, molecular epidemiology, potential risks

## Abstract

*Bartonella bovis* is an emerging ruminant pathogen with zoonotic potential. Although various *Bartonella* species have been detected in animals in Thailand, none have been reported in dairy cattle. We investigated the occurrence and genetic characteristics of *Bartonella* spp. in dairy cattle from central Thailand and examined associations with farm management practices. Blood samples were collected from 522 cattle on 43 farms in four provinces and examined for *Bartonella* DNA by PCR targeting the *gltA*, *rpoB*, and *ribC* genes. Sequencing, phylogenetic analyses, and nucleotide sequence type (ntST) network analyses were also performed. Risk factors were assessed using Fisher’s exact test and multivariable logistic regression. *Bartonella* DNA was detected in 4.21% of samples, with all positives identified as *B. bovis*. Univariable analysis showed significantly higher prevalence in small/medium‐scale farms and separate feeding systems. However, in the multivariable model, farm size showed a strong trend toward association with *B. bovis* detection, although the association did not reach statistical significance. The dominant *gltA* sequence type (ntST04) was detected across all farm sizes and feeding practices, whereas a peripheral variant (ntST02) differed by nine nucleotide substitutions from a ked‐associated *Bartonella* sequence (GenBank ID: OQ716819). Both *rpoB* and *ribC* networks revealed seven distinct groups that were closely related to the *B. bovis* reference sequences, with nucleotide identities of 99.42%–100% and 99.41%–100%, respectively. This study provides new molecular evidence of *B. bovis* circulation in the Thai dairy cattle population, highlighting the need for improved surveillance and biosecurity, particularly in small‐scale operations, to mitigate potential zoonotic risks within a One Health framework.

## 1. Introduction

The genus *Bartonella* comprises facultative intracellular bacteria that infect a wide range of mammalian hosts, including humans, domestic animals, and wildlife [1]. Transmission primarily occurs through arthropod vectors, including fleas, sand flies, lice, and potentially ticks [2–5]. The public health significance of *Bartonella* lies in its zoonotic potential as several species are recognized human pathogens capable of causing a broad range of clinical manifestations, from localized lymphadenopathy to severe systemic infections such as endocarditis and bacteremia [6]. Many animals serve as reservoirs for *Bartonella* transmission, causing disease in cats, dogs, and cattle [7–9].

Among the various *Bartonella* spp., the species prevalent in cattle is *B. bovis* [8, 10]. However, the full spectrum of diseases associated with *B. bovis* infection in cattle remains unclear, with some studies suggesting that it is linked to endocarditis and reproductive disorders [8, 11, 12]. Emerging evidence suggests that *B. bovis* can infect humans, as evidenced by its detection and isolation from a veterinarian in Mexico [13]. Additionally, related species such as *B. schoenbuchensis* and *B. melophagi*, primarily found in domestic cattle and sheep and their ectoparasites have also been reported in human cases [14, 15]. Thus, the potential zoonotic nature of ruminant‐associated *Bartonella* spp. highlights the need for further epidemiological studies to assess its impact on public health.

In Thailand, *Bartonella* spp. have been detected in various animal hosts, with *B. tamiae*, *B. henselae*, and *B. vinsonii* subsp. *arupensis* detected or isolated from human cases [16–20]. Nevertheless, human bartonellosis associated with ruminants has never been reported in Thailand [21–23]. *Bartonella* DNA has been detected in domestic cattle, captive Eld’s deer, and their ectoparasites [24–27], and seropositivity to various *Bartonella* spp. has been reported in water buffaloes [28]; however, comprehensive epidemiological data on *Bartonella* spp. in Thai dairy populations remain limited. Dairy cattle were selected as the primary focus of this study because they generally have longer productive lifespans than beef cattle, potentially resulting in prolonged exposure to environmental vectors and vector‐borne pathogens [12, 29]. In addition, dairy production systems involve intensive daily management and frequent human–animal contact during milking and routine husbandry practices, which may increase the risk of pathogen transmission and zoonotic exposure [6, 30]. Given the expansion of the dairy industry in Thailand and the potential health impacts of *Bartonella* infections in both animals and humans, systematic surveillance and molecular characterization of *Bartonella* in ruminants are important for improving our understanding of its epidemiology and associated risks.

Investigating the prevalence and distribution of *Bartonella* spp. in dairy cattle populations is critical for evaluating impacts on animal and public health. Documenting its presence in dairy cattle in Thailand could enhance the understanding of its transmission patterns and inform future studies on zoonotic potential. This study aims to detect and characterize *Bartonella* infections in dairy cattle in the central region of Thailand while assessing potential risks. Our findings contribute to a more comprehensive understanding of *Bartonella* dynamics within the livestock sector and highlight its potential implications for public health.

## 2. Materials and Methods

### 2.1. Sample Collection

**Figure 1:**
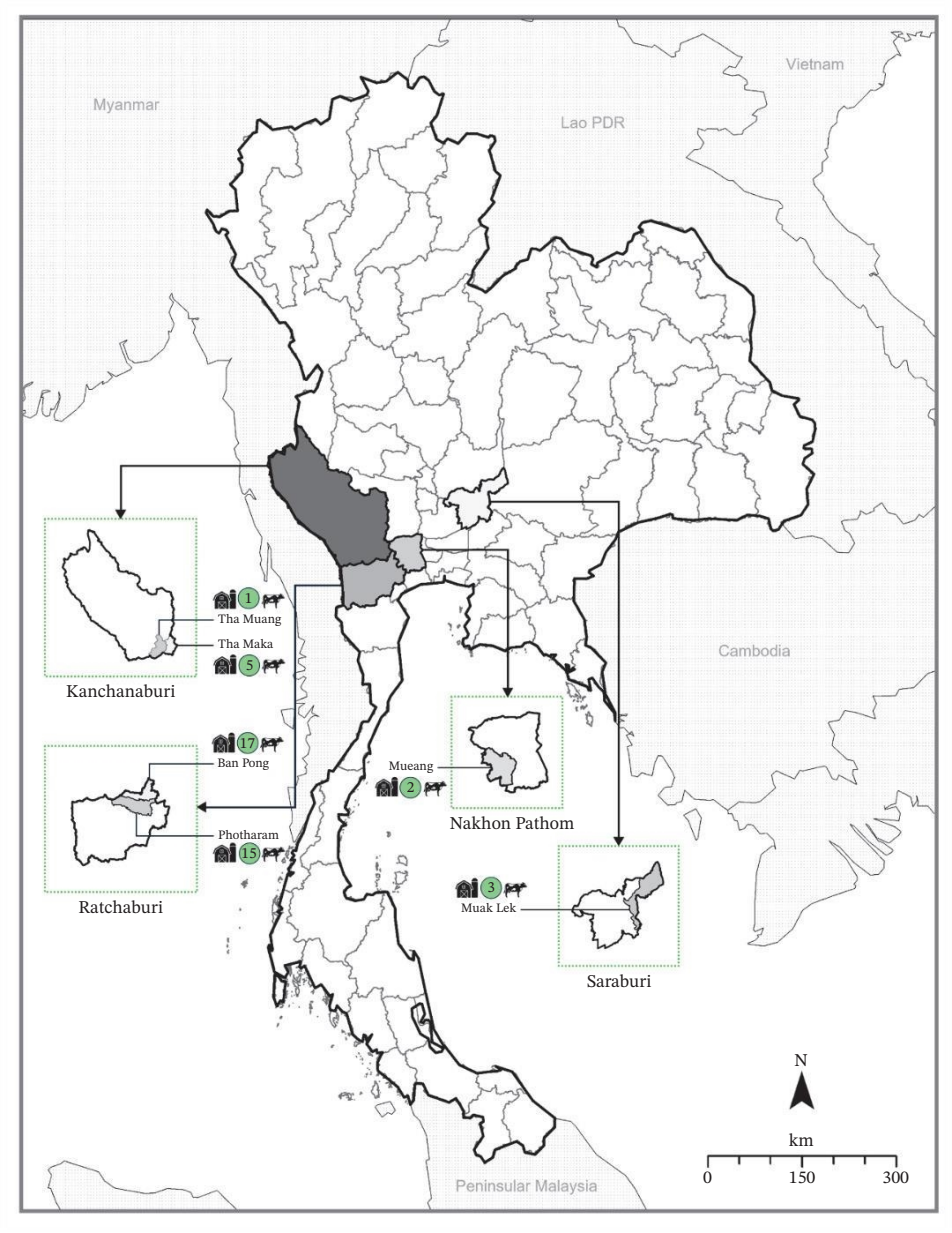
Location of sampling sites. Nakhon Pathom, Saraburi, Kanchanaburi, and Ratchaburi provinces, Thailand, are shown in colored areas.

Blood samples were collected in May 2024 from lactating female Holstein–Friesian crossbred dairy cattle raised on farms in Nakhon Pathom, Saraburi, Kanchanaburi, and Ratchaburi provinces, Thailand (Figure 1). The sample size was calculated based on the methodology described by Thrusfield [31] using the standard formula for estimating prevalence in a large population: *n = Z^2^P* (*1−P*) */ d^2^*, where *n* is the required sample size, *Z* represents the *Z* value for a 95% confidence level (1.96), *P* is the expected prevalence, and *d* is the desired precision. In the absence of prior prevalence data for *Bartonella* spp. In Thai dairy cattle, the expected prevalence was conservatively set at 50% with a precision of 5%, resulting in a minimum required sample size of 384 animals. During the sampling period, all eligible and accessible dairy cattle from participating farms were included in the study, resulting in a final sample size of 522 dairy cattle. In addition to collecting samples, we collected data on farm size, housing conditions, feeding practices, and milking systems. Individual age and body weight data were not recorded during field sampling; therefore, the homogeneity of these characteristics across farms could not be assessed. However, all sampled animals were lactating female Holstein–Friesian crossbred dairy cattle. At the time of sampling, all participating farms were under the supervision of the Veterinary Teaching Hospital at Nong Pho, Ratchaburi, Kasetsart University, and the Faculty of Veterinary Science, Chulalongkorn University. Farm owners granted access to their farms and cooperated with the sample collection. Briefly, 5 mL of blood was collected from the coccygeal vein and placed in EDTA‐coated tubes (BD Vacutainer, Franklin Lakes, New Jersey, USA) and then stored at 4°C in a portable cooler. All samples were transported within 2 days to the Parasitology Unit, Faculty of Veterinary Science, Chulalongkorn University, for further laboratory analysis.

### 2.2. DNA Extraction and PCR Amplification

DNA was extracted from 200 µL of blood using the IndiSpin Pathogen Kit (Indical Bioscience, Leipzig, Germany), following the manufacturer’s instructions. All DNA samples were initially screened for *Bartonella* DNA using PCR targeting the *gltA* gene, which provides high discriminating power for detecting genetic variants. Samples that tested positive for *gltA* were subsequently subjected to PCR amplification of the *rpoB* and *ribC* genes to support robust species‐level identification [32]. All primers used in this study are listed in Table S1. Each PCR reaction contained 1.5 µL of the DNA template, 12.5 µL of 2× PCR buffer (GoTaq Green Master Mix, Promega Corporation, Madison, WI, USA), and 0.75 µL each of forward and reverse primers (10 μM). Sterile distilled water was added to obtain a final reaction volume of 25 µL. The PCR cycles consisted of an initial denaturation at 95°C for 2 min, followed by 40 cycles of denaturation at 95°C for 30 s, gene‐specific annealing, and extension. The annealing temperatures were as follows: 48°C, 30 s for *gltA*; 56°C, 30 s and 59°C, 30 s for the outer and inner *rpoB* reactions, respectively; and 51°C, 1 min for *ribC*. The extension step was performed at 74°C for 1 min, with a final extension at 74°C for 5 min. Template DNA from *B. henselae* and distilled water served as positive and negative controls, respectively. *Bartonella* PCR‐positive products were purified using a GenepHlow Gel/PCR cleanup kit (Geneaid Biotech Ltd., Taipei City, Taiwan) and submitted to U2Bio Co., Ltd. (Seoul, South Korea) for nucleotide sequencing.

### 2.3. DNA Sequencing and Sequence Analysis

All target gene sequences from each PCR‐positive sample were compared to deposited nucleotide sequences in the GenBank database using the NCBI nucleotide BLAST tool. The sequences were validated, aligned, and compared for genetic similarity using the SeqMan and MegAlign software (DNASTAR, Inc., Madison, Wisconsin, USA). The number of nucleotide sequence types (ntSTs) was then analyzed using DnaSP Version 6.12.03 [33]. For each target gene, a representative sequence from each ntST was selected to construct phylogenetic trees using MEGA X with the best‐fit models [34]. Phylogenetic trees were generated using the maximum likelihood (ML) algorithm with the Tamura 3‐parameter model plus gamma distribution (T92 + G) for the *gltA* gene, Tamura–Nei model plus gamma distribution (TN93 + G) for the *rpoB* gene, and the Hasegawa–Kishino–Yano model plus gamma distribution (HKY + G) for the *ribC* gene. Bootstrap support was inferred from 1000 replications. The ntST networks were constructed using the Templeton–Crandall–Sing (TCS) genetic network in PopART Version 1.7 [35, 36].

### 2.4. Statistical Analysis

The relationships between *Bartonella* detection status and farm management factors (farm sizes, housing conditions, feeding practices, and milking systems) were initially analyzed using a two‐tailed Fisher’s exact test. Factors showing significant associations (*p* < 0.05) were further evaluated using multivariable logistic regression to identify independent predictors. All statistical analyses were performed using GraphPad Prism (Version 8.4.2, GraphPad Software, San Diego, California, USA). A *p*‐value < 0.05 was considered statistically significant, and results were reported with 95% confidence intervals. Given the relatively small number of PCR‐positive samples, 95% confidence intervals for adjusted odds ratios (aORs) were estimated using the profile likelihood method, whereas *p*‐values were calculated using the Wald test. Consequently, the statistical significance indicated by the 95% confidence interval and the *p*‐value may differ slightly near the significance threshold. For ease of interpretation, large‐scale farms and total mixed ration (TMR) feeding were designated as the reference categories.

## 3. Results

### 3.1. Sample Distribution and Farm Characteristics

Regarding the animal characteristics, all sampled dairy cattle were female, Holstein–Friesian crossbred, lactating cows. Of the 522 dairy cattle blood samples collected from 43 farms, 20 samples (3.83%) were from two farms in Nakhon Pathom province, 143 samples (27.40%) were from three farms in Saraburi province, 59 samples (11.30%) were from six farms in Kanchanaburi province, and 300 samples (57.47%) were from 32 farms in Ratchaburi province (Table 1).

**Table 1:** Summary of farm management factors and sample distribution (*n* = 43 farms, 522 samples).

Category	Sub‐category	No. of farms (%)	No. of samples (%)
Province	Ratchaburi	32 (74.42%)	300 (57.47%)
	Saraburi	3 (6.98%)	143 (27.40%)
	Kanchanaburi	6 (13.95%)	59 (11.30%)
	Nakhon Pathom	2 (4.65%)	20 (3.83%)
Farm size	Small‐to‐medium (< 50 cattle)	38 (88.37%)	368 (70.50%)
	Large (≥ 50 cattle)	5 (11.63%)	154 (29.50%)
Housing condition	Tie‐stall	36 (83.72%)	337 (64.56%)
	Loose barn	7 (16.28%)	185 (35.44%)
Feeding practice	Separate feeding	36 (83.72%)	341 (65.32%)
	Total mixed ration (TMR)	7 (16.28%)	181 (34.68%)
Milking system	Parallel bucket machine	40 (93.02%)	426 (81.61%)
	Herringbone automatic pipeline	3 (6.98%)	96 (18.39%)

Five farms with 50 or more dairy cattle were classified as large‐scale, whereas 38 farms with fewer than 50 cattle were considered small‐ or medium‐scale. The majority of farms (*n* = 36) practiced tie‐stall housing, while seven farms practiced loose barn housing. Seven farms used a TMR system, whereas the remaining farms used separate feeding systems. Forty farms milked their cows using a parallel bucket milking machine, while the remaining three farms used a herringbone automatic pipeline system (Table 1).

### 3.2. The Prevalence and Associated Risk Factors of *Bartonella* in Dairy Cattle

*Bartonella* was detected in 4.21% (22/522) of the dairy blood samples. Of the positive samples, 21 were obtained from 11 farms in Ratchaburi province (7.00%; 21/300) and one was from one farm in Saraburi province (0.70%; 1/143).

The association between the prevalence of *Bartonella* spp. and farm management practices are summarized in Table 2. The bacterium was detected at a significantly higher rate in cattle from small/medium‐scale farms (5.43%, 20/368) compared to those in large‐scale farms (1.30%, 2/154) (OR = 4.37; 95% CI: 1.16–19.09; *p* = 0.0317). Similarly, it was more prevalent in cattle from farms using separate feeding systems (5.87%, 20/341) than in those using TMR (1.10%, 2/181) (OR = 5.58; 95% CI: 1.45–24.35; *p* = 0.0102). In contrast, housing conditions and milking systems were not significantly associated with *Bartonella* detection. We noted higher prevalence rates in tie‐stall housing (5.34%, 18/337) and farms using bucket milking machines (4.69%, 20/426); however, these associations were not statistically significant (OR = 2.55; 95% CI: 0.87–7.06; *p* = 0.1103 and OR = 2.32; 95% CI: 0.59–10.17; *p* = 0.3976, respectively).

**Table 2:** Association between *Bartonella* spp. prevalence and different farm management practices.

Factors			PCR *Bartonella* spp.		Two‐tailed Fisher’s exact test	
			Positive	Negative	OR (95% CI)	*p*‐Value
Farm size	Small/medium	368	20 (5.43%)	348 (94.57%)	4.37 (1.16–19.09)	0.0317^∗^
	Large	154	2 (1.30%)	152 (98.70%)		
Housing condition	Tie‐stall	337	18 (5.34%)	319 (94.66%)	2.55 (0.87–7.06)	0.1103
	Loose barn	185	4 (2.16%)	181 (97.84%)		
Feeding practice	Separate	341	20 (5.87%)	321 (94.13%)	5.58 (1.45–24.35)	0.0102^∗^
	Total mixed ration	181	2 (1.10%)	179 (98.90%)		
Milking system	Bucket	426	20 (4.69%)	406 (95.31%)	2.32 (0.59–10.17)	0.3976
	Pipeline	96	2 (2.08%)	94 (97.92%)		

^∗^Significant values (*p* < 0.05).

To assess the independent influence of these factors, a multivariable logistic regression model was fitted (Table 3). After adjusting for both variables, the association between separate feeding systems and *Bartonella* prevalence was no longer significant (aOR = 1.38; 95% CI: 0.27–25.19; *p* = 0.7594). Interestingly, the small/medium‐scale farms had an aOR of 4.33 (95% CI: 1.24–27.38) compared to large‐scale farms; however, the statistical significance was slightly above the threshold (*p* = 0.0508) in the multivariable model. The model’s predictive performance was evaluated using the area under the ROC curve (AUC), which was 0.62 (95% CI: 0.51–0.73), indicating limited discriminative ability.

**Table 3:** Multivariable logistic regression model for *Bartonella* spp. infection.

Factors	Coefficient (*β*)	Adjusted OR (95% CI)	*p*‐Value
Farm size (small/medium vs. large)	1.465	4.33 (1.24–27.38)	0.0508
Feeding practice (separate vs. TMR)	0.320	1.38 (0.27–25.19)	0.7594

*Note:* Area under the ROC curve (AUC): 0.62 (95% CI: 0.51–0.73). Variables with *p* < 0.05 in the univariable analysis were included in the model. Large‐scale farms and total mixed ration feeding were used as the reference categories. The 95% confidence intervals were calculated using the profile likelihood method, whereas *p*‐values were obtained using the Wald test.

### 3.3. BLAST and Genetic Analyses of *Bartonella* Detected in Dairy Cattle

For each target gene, we aligned 22 nucleotide sequences and then classified them into ntSTs using DnaSP version 6.12.03. Among the sequences validated as *Bartonella*, the *gltA* sequences (337 bp) were categorized into six ntSTs (Acc. No. PX401255–PX401260), while the *rpoB* (861 bp) and *ribC* (535 bp) sequences were each grouped into seven ntSTs (Acc. No. PX401261–PX401267 and PX401270–PX401276, respectively) (Table 4).

**Table 4:** Nucleotide sequence types (ntSTs) and NCBI BLAST results of the representative ntSTs obtained in this study.

ntSTs	Detected *Bartonella* genes					
	*gltA*		*rpoB*		*ribC*	
	No. of sequences (*N* = 22)	Highest BLAST (% identity)	No. of sequences (*N* = 22)	Highest BLAST (% identity)	No. of sequences (*N* = 22)	Highest BLAST (% identity)
1	7 (PX401255)	*B. bovis*/beef cattle/Israel/KJ909835 (100%) [37]	13 (PX401261)	*B. bovis*/beef cattle/Israel/KJ909808 (99.88%) [37]	6 (PX401270)	*B. bovis*/beef cattle/Israel/KM371091 (99.41%) [unpublished]
2	1 (PX401256)	*Bartonella* sp./ked/Thailand/OQ716819 (97.92%) [27]	3 (PX401262)	*B. bovis*/cattle/Thailand/KF218220 (100%) [24]	4 (PX401271)	*B. bovis*/cattle/Thailand/KF218211 (100%) [24]
3	1 (PX401257)	*B. bovis*/cattle/Malaysia/KR733182 (99.70%) [38]	1 (PX401263)	*B. bovis*/beef cattle/Israel/KJ909808 (99.54%) [37]	5 (PX401272)	*B. bovis*/cattle/Malaysia/KR733191 (100%) [38]
4	11 (PX401258)	*B. bovis*/cattle/Malaysia/KR733182 (100%) [38]	2 (PX401264)	*B. bovis*/cattle/Malaysia/KR733194 (100%) [38]	2 (PX401273)	*B. bovis*/cattle/Guatemala/KF218210 (100%) [24]
5	1 (PX401259)	*B. bovis*/beef cattle/Israel/KJ909835 (99.70%) [37]	1 (PX401265)	*B. bovis*/beef cattle/Israel/KJ909808 (99.54%) [37]	3 (PX401274)	*B. bovis*/cattle/Malaysia/KR733191 (99.81%) [38]
6	1 (PX401260)	*B. bovis*/beef cattle/Israel/KJ909820 (99.70%) [37]	1 (PX401266)	*B. bovis*/beef cattle/Israel/KJ909808 (99.42%) [37]	1 (PX401275)	*B. bovis*/cattle/Thailand/KF218211 (100%) [24]
7	—	—	1 (PX401267)	*B. bovis*/cattle/Malaysia/KR733194 (99.88%) [38]	1 (PX401276)	*B. bovis*/beef cattle/Israel/KM371091 (99.63%) [unpublished]

BLAST analysis of the *gltA* sequences showed that ntST01 and ntST03 to ntST06 are closely related to *B. bovis* isolates or sequences obtained from cattle in various countries (KJ909820, KJ909835, and KR733182), with identity scores of 99.70% to 100%. In contrast, ntST02 is 97.92% similar to *Bartonella* spp. previously detected in keds collected from Eld’s deer in Thailand (OQ716819). All seven ntSTs of the *rpoB* gene are 99.42% to 100% similar to *B. bovis* strains from cattle (KJ909808, KR733194, and KF218220). Similarly, the seven ntSTs of the *ribC* gene are 99.41%–100% similar to *B. bovis* sequences (KF218210, KF218211, KM371091, and KR733191) (Table 4).

For phylogenetic and TCS ntST network analyses, the obtained sequences were truncated to match reference data, resulting in final lengths of 337 bp for *gltA*, 779 bp for *rpoB*, and 510 bp for *ribC*. The results of phylogenetic analysis are consistent with the BLAST sequence similarity results. The phylogenetic tree based on the *Bartonella gltA* gene shows ntST01 and ntST03 to ntST06 clustering within the *B. bovis* clade, while ntST02 is grouped with the clade of ked‐associated *Bartonella* spp. (Figure 2A). Meanwhile, the *rpoB* and *ribC* phylogenetic trees show all *Bartonella* sequences obtained in this study clustering within the *B. bovis* group (Figures 3A and 4A).

Figure 2ML tree and ntST networks of the *gltA* gene of *Bartonella* sequences (337 bp). The ML tree was computed with the T92 + G model. The phylogenetic relationships are shown among sequences obtained in this study (indicated by black dots) and reference sequences retrieved from the GenBank database (A). TCS ntST networks of *gltA* sequences. Nodes labeled ntST01 to ntST06 represent sequences obtained in this study, whereas the remaining nodes represent reference sequences retrieved from GenBank and used in the phylogenetic tree. The size of each circle/node is proportional to the frequency (number of sequences) of that ntST. The color of the node represents the farm size (B) and feeding practice (C). Each perpendicular line/hash mark on the connecting line represents one mutational step (one base‐pair change) between two ntSTs. “N/A” refers to the reference sequences for which epidemiological data (farm size and feeding practice) were not applicable.

**Figure:**
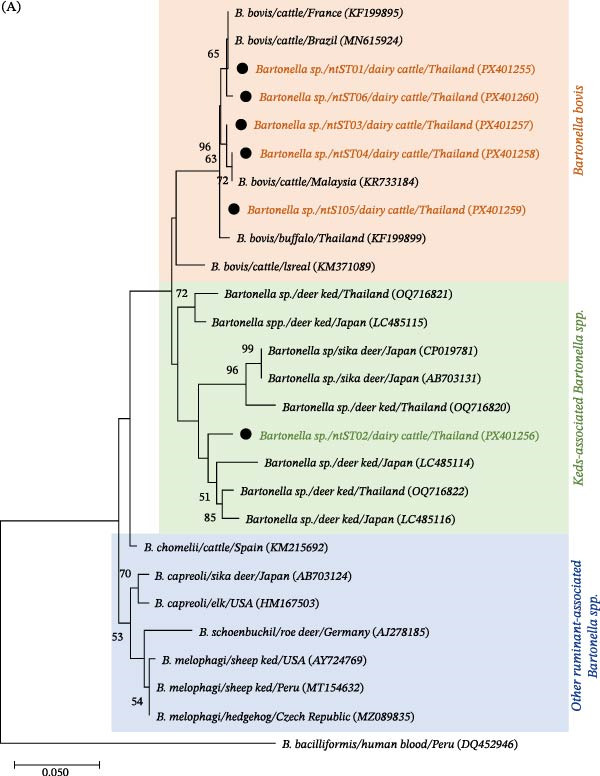

**Figure:**
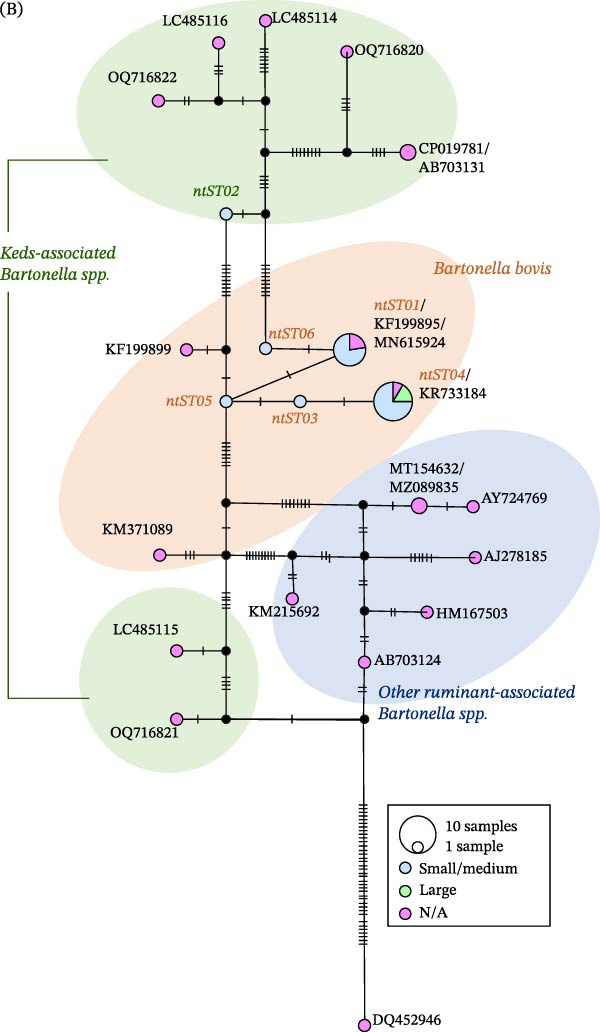

**Figure:**
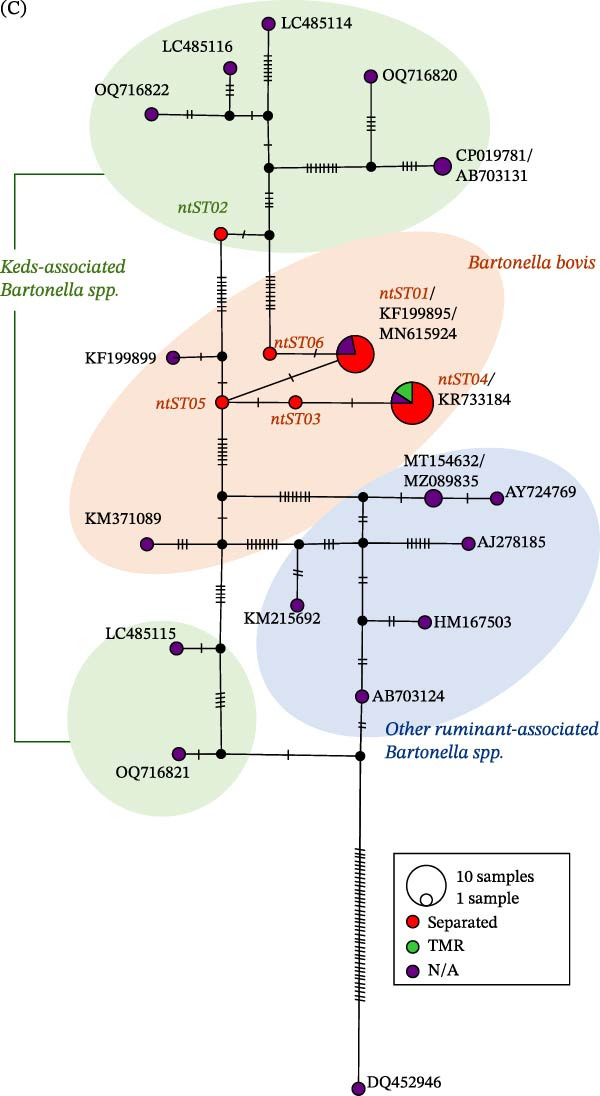

Figure 3ML tree and ntST networks of the *rpoB* gene of *Bartonella* sequences (779 bp). The ML tree was computed with the TN93 + G model. The phylogenetic relationships are shown among sequences obtained in this study (indicated by black dots) and reference sequences retrieved from the GenBank database (A). TCS ntST networks of *rpoB* sequences. Nodes labeled ntST01 to ntST07 represent sequences obtained in this study, whereas the remaining nodes represent reference sequences retrieved from GenBank and used in the phylogenetic tree. The size of each circle/node is proportional to the frequency (number of sequences) of that ntST. The color of the node represents the farm size (B) and feeding practice (C). Each perpendicular line/hash mark on the connecting line represents one mutational step (one base‐pair change) between two ntSTs. “N/A” refers to the reference sequences for which epidemiological data (farm size and feeding practice) were not applicable.

**Figure:**
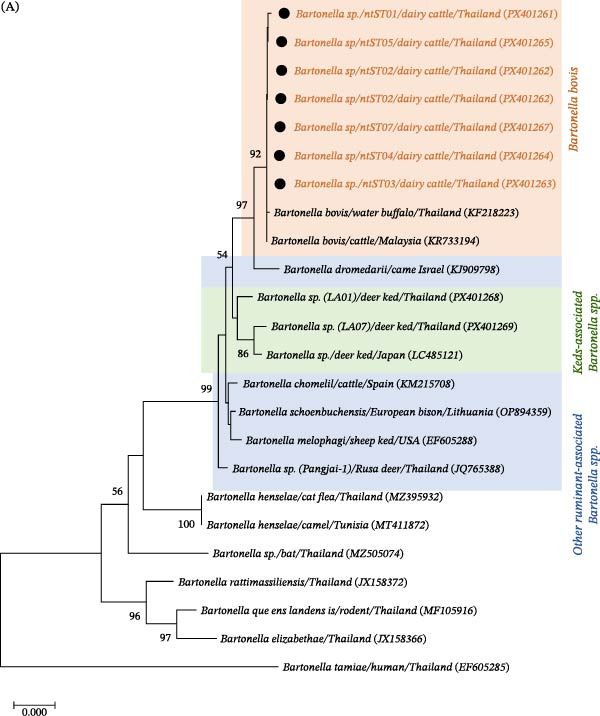

**Figure:**
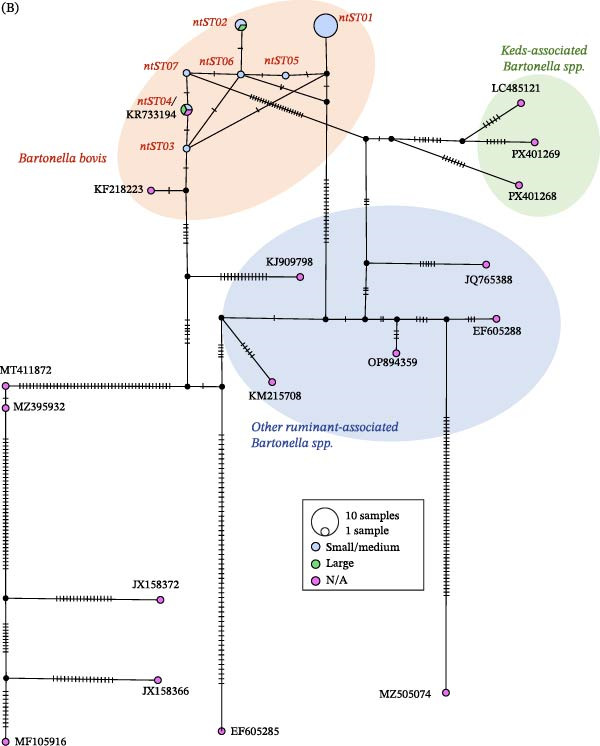

**Figure:**
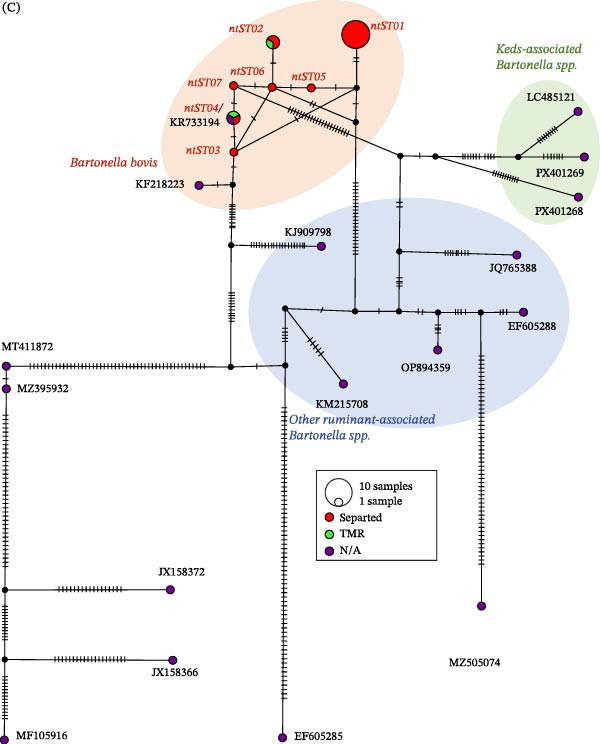

Figure 4ML tree and ntST networks of the *ribC* gene of *Bartonella* sequences (510 bp). The ML tree was computed with the HKY + G model. The phylogenetic relationships are shown among sequences obtained in this study (indicated by black dots) and reference sequences retrieved from the GenBank database (A). TCS ntST networks of *ribC* sequences. Nodes labeled ntST01 to ntST07 represent sequences obtained in this study, whereas the remaining nodes represent reference sequences retrieved from GenBank and used in the phylogenetic tree. The size of each circle/node is proportional to the frequency (number of sequences) of that ntST. The color of the node represents the farm size (B) and feeding practice (C). Each perpendicular line/hash mark on the connecting line represents one mutational step (one base‐pair change) between two ntSTs. “N/A” refers to the reference sequences for which epidemiological data (farm size and feeding practice) were not applicable.

**Figure:**
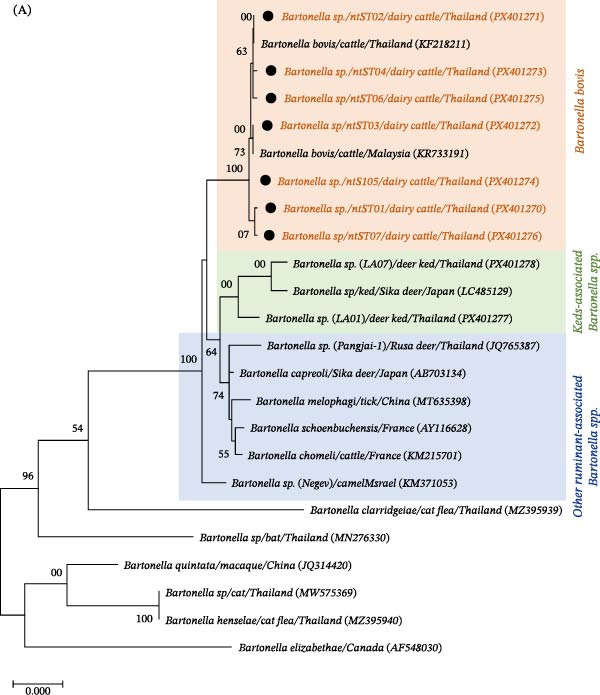

**Figure:**
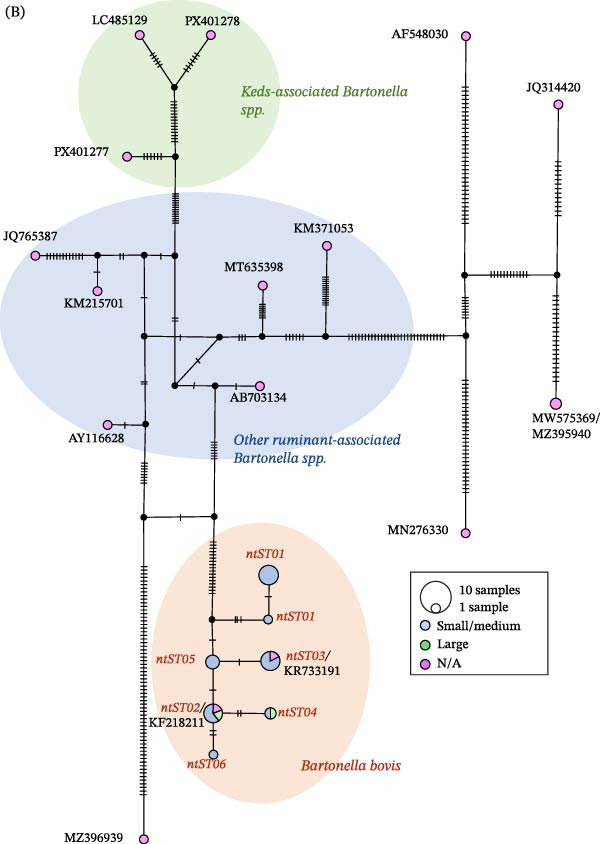

**Figure:**
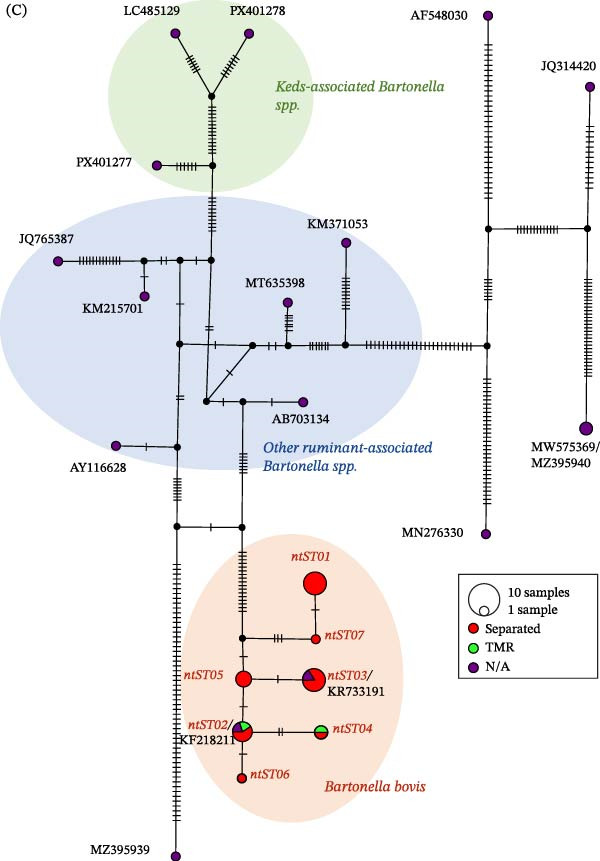

To investigate the relationship between genetic types and potential epidemiological factors driving the distribution of *Bartonella* spp., we constructed TCS ntST networks for *gltA*, *rpoB*, and *ribC*. The analysis focused on two significant farm management variables: farm size (small/medium vs. large) and feeding practice (separate vs. TMR). The TCS ntST network of *gltA* sequences consists of six distinct groups (ntST01–ntST06) (Figure 2B,C), dominated by ntST04, which was detected across all farm sizes (small/medium and large) and both feeding practices (separate and TMR). Notably, ntST02 differed from the closest ked‐associated *Bartonella* sequence (GenBank Accession No. OQ716819) by nine nucleotide substitutions and was positioned peripherally relative to the other ntSTs.

The TCS ntST network of the *rpoB* gene consisted of seven distinct groups (ntST01–ntST07) (Figure 3B,C), all of which were closely related to reference sequences of *B. bovis* (KR733194 and KF218223). All ntSTs were detected in samples from small‐/medium‐scale farms using a separate feeding system. However, ntST02 and ntST04 were also detected in samples from large farms employing the TMR feeding system. The ntST network of the *ribC* gene consisted of groups that were distributed across farm characteristics, with no clearly dominant ntST (Figure 4B,C).

## 4. Discussion

We estimate the overall prevalence of *B. bovis* in dairy cattle at 4.21%, which is low compared to the wide range of prevalence rates that have been reported globally (15% to over 50%) [12, 37, 39, 40]. Such variation in prevalence likely reflects differences in geographic location, herd management, and, importantly, detection methods. Unlike studies that employ culture‐based enrichment [12, 37] or serological prescreening [39], which often report higher prevalence rates, our study used direct PCR from whole blood. This approach provides a conservative estimate of the molecular prevalence of current bacteremia, although it may underestimate infections due to the intermittent nature of *Bartonella* bacteremia. Nevertheless, the use of universal primers and the clear sequencing chromatograms supported reliable species‐level identification; however, low‐abundance mixed infections or coexisting variants could not be completely excluded. Dairy and other domestic cattle may host a diverse range of other *Bartonella* spp., including *B. chomelii* (found in Algeria, Spain, and Georgia) and *B. schoenbuchensis* (associated with deer but also found in European cattle and flies) [14, 15, 29, 39, 41], indicating that cattle may host a diverse range of *Bartonella* spp. Their prevalence and distribution are likely shaped by regional ecological factors, animal husbandry practices, and vector populations. Such diversity underscores the importance of context‐specific assessment of risk factors to inform surveillance strategies.

Our univariable analysis revealed significant associations between *Bartonella* detection and farm management practices. Specifically, the bacterium tended to be detected more in cattle from small/medium farms than in those from large‐scale operations. This finding is consistent with the known greater prevalence of the pathogen in smaller cattle farms due to less stringent biosecurity and limited resources for addressing vector control and herd health management [42–45]. Similarly, *B. bovis* was more prevalent in separate feeding systems, suggesting that feed handling and hygiene may influence the risk of infection. In many cases, manual handling and the distribution of forage in open troughs can lead to the accumulation of spilled feed and organic residues. When mixed with manure and moisture, these materials create favorable breeding conditions for hematophagous flies such as *Stomoxys* spp., *Haematobia* spp., and *Hippobosca* spp., which have been reported as potential vectors of *Bartonella* species in cattle [39, 46–48]. These flies are widely distributed and often show high seasonal abundance in livestock environments in Thailand. For example, *Stomoxys* spp. are prevalent across multiple regions of the country [49], while substantial populations of Tabanidae have been documented in cattle farming areas [50, 51].

However, when these potential risk factors were evaluated simultaneously in the multivariable logistic regression model, the significance of the feeding system was lost. This suggests that the influence of feeding practices may be confounded by farm size; for instance, separate feeding might be more prevalent in small‐scale farms [52]. Farm size retained a strong but non‐significant association after adjustment, whereas feeding practice was not associated with detection in the multivariable model. These findings should be interpreted cautiously because of the limited number of positive samples and the uneven distribution of samples across farm categories [40]. The higher risk observed in smaller farms likely reflects a combination of environmental and husbandry factors, including animal confinement and less effective vector control [43, 44]. For instance, tie‐stall housing and the use of bucket milking systems, which are more common in small‐scale operations, are often associated with higher densities of hematophagous flies and ectoparasites due to challenges in maintaining consistent environmental hygiene [53, 54]. Collectively, these findings suggest that the improved management standards and more systematic vector control programs typically implemented in larger farms may provide greater protection against vector‐borne infections [55].

This study demonstrates the utility of multilocus markers for accurate species identification, evaluated through their respective discriminating powers as defined by the *Bartonella* paradigm [32]. The *gltA* gene, known for its high resolution in *Bartonella* systematics, enabled the detection of subtle genetic variations among the sequences. Notably, one sequence type showed close similarity to a *Bartonella* variant previously reported in wildlife; however, the sequence similarity remained well within the established species‐definition threshold for *gltA*. These findings were further supported by the more conserved *rpoB* and *ribC* loci, which consistently placed all sequences within the *B. bovis* clade.

Multilocus sequence analysis supported the identification of the detected *Bartonella* as *B. bovis*. One *gltA* sequence type was genetically related to a *Bartonella* sequence previously detected in keds collected from captive Eld’s deer in a wildlife sanctuary [27]. This finding, together with previous reports of *Bartonella* in Rusa deer from Ratchaburi [25] and its detection in ectoparasites collected from bats in the same province [56], highlights the widespread circulation of *Bartonella* spp. in the area. It also suggests a potential role of wild animals and their associated vectors as reservoirs or sources of infection for cattle at the wildlife–livestock interface.

The results of the ntST network analysis support epidemiological observations, i.e., the dominant group, *gltA* ntST04, was present across samples from both small/medium and large farms, as well as both separate and TMR feeding systems. This indicates its widespread circulation in the region, regardless of the farm scale and management. This genetic homogeneity suggests that while farm management practices may influence the prevalence, the most widely circulating strains are not strictly constrained by specific management barriers. These findings indicate that several genetic variants were detected across different farm management systems. Further studies incorporating ectoparasite sampling are needed to clarify their transmission routes and regional distribution.

Several limitations of this study should be acknowledged. Because our findings rely solely on molecular assessment without bacterial isolation of *B. bovis*, potential PCR amplification biases cannot be entirely ruled out. Furthermore, direct sequencing of PCR products may not fully capture mixed infections or multiple coexisting variants of *B. bovis* within a single host, which warrants further investigation using cloning techniques or next‐generation sequencing. Tick infestation was not systematically assessed or quantified during sampling; therefore, no conclusions regarding the tick burden could be drawn. Given the potential role of ectoparasites, including ticks, lice, and keds, in the transmission of *Bartonella* spp., future research should investigate the presence of *Bartonella*, including *B. bovis*, in ectoparasites collected directly from dairy cattle in Thailand. The zoonotic potential of *B. bovis* remains a growing concern, especially among veterinarians, farmers, and abattoir workers due to occupational exposure [13, 14]. Assessing and mitigating potential zoonotic risks require surveillance strategies such as those espoused by the One Health‐based approach, i.e., encompassing human, animal, and environmental health domains.

## 5. Conclusion

Our study provides molecular evidence of *B. bovis* in Thai dairy cattle and supports continued surveillance and a One Health‐based approach to better understand its ecology, transmission dynamics, and potential zoonotic implications. Genetic characterization via ntST networks confirmed the circulation of highly successful *B. bovis* strains across all farm management types while also suggesting a potential genetic link to strains found in wildlife. The findings also highlight the importance of farm management and hygiene, particularly in small‐scale operations, where the bacterium was more prevalent. Enhancing awareness, education, and biosecurity practices among farm workers is essential to reduce infection risk and prevent cross‐species transmission.

## Author Contributions

**Wittawat Wechtaisong and Wisa Tiyamanee**: methodology. **Wittawat Wechtaisong**: investigation, data curation, writing – original draft. **Kritsada Thongmeesee**: visualization. **Chalida Sri-in and Aung Aung:** validation. **Satoru Konnai**: conceptualization. **Sonthaya Tiawsirisup**: supervision, funding acquisition, project administration. **Kritsada Thongmeesee** **, Chalida Sri-in, Aung Aung, Wisa Tiyamanee, Satoru Konnai, and Sonthaya Tiawsirisup:** writing – review and editing.

## Funding

This work was supported by the Thailand Science Research and Innovation Fund Chulalongkorn University 2026 (Grant FOOD_FF_69_092_3100_008) and the Chulalongkorn University Center of Excellence (Grant CE69_033_3100_007).

## Disclosure

All authors contributed to the article and approved the submitted version.

## Ethics Statement

This study was approved by the Chulalongkorn University Animal Care and Use Committee (IACUC Protocol Number 2431064) and the CUVET Institutional Biosafety Committee (IBC Protocol Number 2431024).

## Conflicts of Interest

The authors declare no conflicts of interest.

## Supporting Information

Additional supporting information can be found online in the Supporting Information section.

## Supporting information


**Supporting Information** Associated with this article can be found in the online version.

## Data Availability

Representative sequences generated in this study were deposited in GenBank under Accession Numbers PX401255–PX401260 for *gltA*, PX401261–PX401267 for *rpoB*, and PX401270–PX401276 for *ribC*.

## References

[1] Breitschwerdt E. B. , Bartonellosis, One Health and All Creatures Great and Small, Veterinary Dermatology. (2017) 28, no. 1, 96–e21, 10.1111/vde.12413. 28133871

[2] Angelakis E. , Billeter S. A. , Breitschwerdt E. B. , Chomel B. B. , and Raoult D. , Potential for Tick-borne Bartonelloses, Emerging Infectious Diseases. (2010) 16, no. 3, 385–391, 10.3201/eid1603.081685. 20202411 PMC3322042

[3] Chomel B. B. , Kasten R. W. , and Floyd-Hawkins K. , et al.Experimental Transmission of *Bartonella henselae* by the Cat Flea, Journal of Clinical Microbiology. (1996) 34, no. 8, 1952–1956, 10.1128/jcm.34.8.1952-1956.1996. 8818889 PMC229161

[4] Ellis B. A. , Rotz L. D. , and Leake J. A. , et al.An Outbreak of Acute Bartonellosis (Oroya Fever) in the Urubamba Region of Peru, 1998, The American Journal of Tropical Medicine and Hygiene. (1999) 61, no. 2, 344–349, 10.4269/ajtmh.1999.61.344. 10463692

[5] Swift H. F. , Trench Fever, Archives of Internal Medicine. (1920) 26, no. 1, 76–98, 10.1001/archinte.1920.00100010079006.

[6] Cheslock M. A. and Embers M. E. , Human Bartonellosis: An Underappreciated Public Health Problem?, Tropical Medicine and Infectious Disease. (2019) 4, no. 2, 10.3390/tropicalmed4020069, 69. 31010191 PMC6630881

[7] Chomel B. B. and Kasten R. W. , Bartonellosis, An Increasingly Recognized Zoonosis, Journal of Applied Microbiology. (2010) 109, no. 3, 743–750, 10.1111/j.1365-2672.2010.04679.x. 20148999

[8] Maillard R. , Petit E. , and Chomel B. , et al.Endocarditis in Cattle Caused by *Bartonella bovis* , Emerging Infectious Diseases. (2007) 13, no. 9, 1383–1385, 10.3201/eid1309.070236. 18252116 PMC2857289

[9] Pultorak E. L. , Linder K. , Maggi R. G. , Balakrishnan N. , and Breitschwerdt E. B. , Prevalence of *Bartonella* spp. in Canine Cutaneous Histiocytoma, Journal of Comparative Pathology. (2015) 153, no. 1, 14–21, 10.1016/j.jcpa.2015.04.001. 25980841

[10] Bermond D. , Boulouis H.-J. , and Heller R. , et al. *Bartonella bovis*, Bermond, Etal, sp. nov. and *Bartonella capreoli* sp. nov., Isolated From European Ruminants, International Journal of Systematic and Evolutionary Microbiology. (2002) 52, no. 2, 383–390, 10.1099/00207713-52-2-383. 11931146

[11] Erol E. , Jackson C. , Bai Y. , Sells S. , Locke S. , and Kosoy M. , *Bartonella bovis* Isolated From a Cow With Endocarditis, Journal of Veterinary Diagnostic Investigation. (2013) 25, no. 2, 288–290, 10.1177/1040638713477408. 23512923

[12] Maillard R. , Grimard B. , and Chastant-Maillard S. , et al.Effects of Cow Age and Pregnancy on *Bartonella* Infection in a Herd of Dairy Cattle, Journal of Clinical Microbiology. (2006) 44, no. 1, 42–46, 10.1128/JCM.44.1.42-46.2006. 16390945 PMC1351957

[13] Gamboa-Prieto J. , Cruz-Romero A. , and Jimenez-Hernandez J. A. , et al.Detection of, *Bartonella bovis*, DNA in Blood Samples From a Veterinarian in Mexico, Revista do Instituto de Medicina Tropical de São Paulo. (2023) 65, 10.1590/s1678-9946202365062, e62. 38055380 PMC10703501

[14] Maggi R. G. , Kosoy M. , Mintzer M. , and Breitschwerdt E. B. , Isolation of, *Candidatus*, Bartonella Melophagi From Human Blood, Emerging Infectious Diseases. (2009) 15, no. 1, 66–68, 10.3201/eid1501.081080. 19116054 PMC2660712

[15] Vayssier-Taussat M. , Moutailler S. , and Femenia F. , et al.Identification of Novel Zoonotic Activity of *Bartonella* spp., France, Emerging Infectious Diseases. (2016) 22, no. 3, 457–462, 10.3201/eid2203.150269. 26885624 PMC4766919

[16] Billeter S. A. , Sangmaneedet S. , and Kosakewich R. C. , et al. *Bartonella* Species in Dogs and Their Ectoparasites From Khon Kaen Province, Thailand, The Southeast Asian Journal of Tropical Medicine and Public Health. (2012) 43, no. 5, 1186–1192. 23431825

[17] Pangjai D. , Nimsuphan B. , and Petkanchanapong W. , et al.First Report of Three Novel *Bartonella* Species Isolated in Rodents and Shrews From Nine Provinces of Thailand, Veterinary World. (2022) 15, no. 7, 1624–1631, 10.14202/vetworld.2022.1624-1631. 36185510 PMC9394139

[18] Poofery J. , Narapakdeesakul D. , and Riana E. , et al.Molecular Identification and Genetic Diversity of *Bartonella* spp. in 24 Bat Species From Thailand, Transboundary and Emerging Diseases. (2022) 69, no. 4, e717–e733, 10.1111/tbed.14389. 34755483

[19] Saengsawang P. , Kaewmongkol G. , and Inpankaew T. , Molecular Detection of *Bartonella* spp. and Hematological Evaluation in Domestic Cats and Dogs From Bangkok, Thailand, Pathogens. (2021) 10, no. 5, 10.3390/pathogens10050503, 503. 33922245 PMC8146774

[20] Saengsawang P. , Pangjai D. , Kaewmongkol G. , and Inpankaew T. , Detection of Antibodies Against Three Zoonotic Bartonella spp. and Cross-Reactivity Among Species and *Coxiella burnetii* in Dogs and Cats From Central Thailand, Comparative Immunology, Microbiology and Infectious Diseases. (2022) 81, 10.1016/j.cimid.2021.101743, 101743. 34942506

[21] Bai Y. , Kosoy M. Y. , and Diaz M. H. , et al. *Bartonella vinsonii* subsp. *arupensis* in Humans, Thailand, Emerging Infectious Diseases. (2012) 18, no. 6, 989–991, 10.3201/eid1806.111750. 22607728 PMC3358162

[22] Kosoy M. , Bai Y. , and Sheff K. , et al.Identification of Bartonella Infections in Febrile Human Patients From Thailand and Their Potential Animal Reservoirs, The American Society of Tropical Medicine and Hygiene. (2010) 82, no. 6, 1140–1145, 10.4269/ajtmh.2010.09-0778. PMC2877425 20519614

[23] Paitoonpong L. , Chitsomkasem A. , and Chantrakooptungool S. , et al. *Bartonella henselae*: First Reported Isolate in a Human in Thailand, The Southeast Asian Journal of Tropical Medicine and Public Health. (2008) 39, no. 1, 123–129. 18567451

[24] Bai Y. , Malania L. , and Castillo D. A. , et al.Global Distribution of *Bartonella* Infections in Domestic Bovine and Characterization of *Bartonella bovis* Strains Using Multi-Locus Sequence Typing, PLoS ONE. (2013) 8, no. 11, 10.1371/journal.pone.0080894, e80894. 24278342 PMC3836770

[25] Pangjai D. , Intachinda S. , and Maruyama S. , et al.Isolation and Phylogenetic Analysis of *Bartonella* Species From Rusa Deer (*Rusa timorensis*) in Thailand, Comparative Immunology, Microbiology and Infectious Diseases. (2018) 56, 58–62, 10.1016/j.cimid.2017.12.005. 29406284

[26] Promrangsee C. , Khositharattanakool P. , and Somwang P. , et al.The Prevalence of *Bartonella* Bacteria in Cattle Lice Collected From Three Provinces of Thailand, Insects. (2019) 10, no. 6, 10.3390/insects10060152, 152. 31142009 PMC6628184

[27] Wechtaisong W. , Sri-In C. , and Thongmeesee K. , et al.Diversity of *Anaplasma* and Novel *Bartonella* Species in *Lipoptena fortisetosa* Collected From Captive Eld’s Deer in Thailand, Frontiers in Veterinary Science. (2023) 10, 10.3389/fvets.2023.1247552, 1247552. 37781280 PMC10538998

[28] Boonmar S. , Saengsawang S. , and Mitsuwan W. , et al.The First Report of the Seroprevalence of Antibodies Against *Bartonella* spp. in Water Buffaloes (*Bubalus bubalis*) From South Thailand, Veterinary World. (2021) 14, no. 12, 3144–3148, 10.14202/vetworld.2021.3144-3148. 35153405 PMC8829419

[29] Antequera-Gómez M. L. , Lozano-Almendral L. , and Barandika J. F. , et al. *Bartonella chomelii* Is the Most Frequent Species Infecting Cattle Grazing in Communal Mountain Pastures in Spain, Applied and Environmental Microbiology. (2015) 81, no. 2, 623–629, 10.1128/AEM.03159-14. 25381240 PMC4277576

[30] Breitschwerdt E. B. and Kordick D. L. , *Bartonella* Infection in Animals: Carriership, Reservoir Potential, Pathogenicity, and Zoonotic Potential for Human Infection, Clinical Microbiology Reviews. (2000) 13, no. 3, 428–438, 10.1128/CMR.13.3.428. 10885985 PMC88941

[31] Thrusfield M. , Surveys, Veterinary Epidemiology, 2018, John Wiley & Sons Ltd, 270–295.

[32] Scola B. L. , Zeaiter Z. , Khamis A. , and Raoult D. , Gene-Sequence-Based Criteria for Species Definition in Bacteriology: The *Bartonella* Paradigm, Trends in Microbiology. (2003) 11, no. 7, 318–321, 10.1016/S0966-842X(03)00143-4. 12875815

[33] Rozas J. , Ferrer-Mata A. , and Sánchez-DelBarrio J. C. , et al.DnaSP6: DNA Sequence Polymorphism Analysis of Large Data Sets, Molecular Biology and Evolution. (2017) 34, no. 12, 3299–3302, 10.1093/molbev/msx248. 29029172

[34] Kumar S. , Stecher G. , Li M. , Knyaz C. , Tamura K. , and Battistuzzi F. U. , MEGA X: Molecular Evolutionary Genetics Analysis Across Computing Platforms, Molecular Biology and Evolution. (2018) 35, no. 6, 1547–1549, 10.1093/molbev/msy096. 29722887 PMC5967553

[35] Clement M. , Snell Q. , and Walker P. , et al.TCS: Estimating Gene Genealogies, *Proceedings 16th International Parallel and Distributed Processing Symposium*, 2002, IEEE, 0184–0184.

[36] Leigh J. W. , Bryant D. , and Nakagawa S. , POPART: Full-Feature Software for Haplotype Network Construction, Methods in Ecology and Evolution. (2015) 6, no. 9, 1110–1116, 10.1111/2041-210X.12410.

[37] Rudoler N. , Rasis M. , Sharir B. , Novikov A. , Shapira G. , and Giladi M. , First Description of *Bartonella bovis* in Cattle Herds in Israel, Veterinary Microbiology. (2014) 173, no. 1-2, 110–117, 10.1016/j.vetmic.2014.07.006. 25096531

[38] Kho K.-L. , Koh F.-X. , Jaafar T. , Nizam Q. N. H. , and Tay S.-T. , Prevalence and Molecular Heterogeneity of *Bartonella bovis* in Cattle and *Haemaphysalis bispinosa* Ticks in Peninsular Malaysia, BMC Veterinary Research. (2015) 11, no. 1, 10.1186/s12917-015-0470-1, 153. 26179499 PMC4502507

[39] Boularias G. , Azzag N. , and Gandoin C. , et al. *Bartonella bovis* and *Bartonella chomelii* Infection in Dairy Cattle and Their Ectoparasites in Algeria, Comparative Immunology, Microbiology and Infectious Diseases. (2020) 70, 10.1016/j.cimid.2020.101450, 101450. 32126432

[40] Secato C. T. , Matos C. A. , and Lee D. A. B. , et al.First Molecular Evidence of *Bartonella* spp. and Hemoplasmas in Cattle From Mozambique, Tropical Animal Health and Production. (2026) 58, no. 2, 10.1007/s11250-026-04877-2, 70. 41603984 PMC12852233

[41] Gutiérrez R. , Cohen L. , and Morick D. , et al.Identification of Different *Bartonella* Species in the Cattle Tail Louse (*Haematopinus quadripertusus*) and in Cattle Blood, Applied and Environmental Microbiology. (2014) 80, no. 17, 5477–5483, 10.1128/AEM.01409-14. 24973066 PMC4136086

[42] Moumouni P. F. A. , Galon E. M. , and Tumwebaze M. A. , et al.Tick-Borne Pathogen Detection and Its Association With Alterations in Packed Cell Volume of Dairy Cattle in Thailand, Animals. (2023) 13, no. 18, 10.3390/ani13182844, 2844. 37760244 PMC10525745

[43] Ferreira J. S. , Baccili C. C. , and Nemoto B. S. , et al.Biosecurity Practices in the Dairy Farms of Southern Brazil, Frontiers in Veterinary Science. (2024) 11, 10.3389/fvets.2024.1326688, 1326688. 38601907 PMC11004291

[44] Heylen D. J. A. , Kumsa B. , and Kimbita E. , et al.Tick-Borne Pathogens and Body Condition of Cattle in Smallholder Rural Livestock Production Systems in East and West Africa, Parasites & Vectors. (2023) 16, no. 1, 10.1186/s13071-023-05709-0, 117. 36998091 PMC10064580

[45] Pozo P. , Isla J. , Asiain A. , Navarro D. , and Gortázar C. , Contribution of Herd Management, Biosecurity, and Environmental Factors to the Risk of Bovine Tuberculosis in a Historically Low Prevalence Region, Animal. (2024) 18, no. 3, 10.1016/j.animal.2024.101105, 101105. 38417216

[46] Baldacchino F. , Muenworn V. , and Desquesnes M. , et al.Transmission of Pathogens by *Stomoxys* Flies (Diptera, Muscidae): A Review, Parasite. (2013) 20, 10.1051/parasite/2013026, 26. 23985165 PMC3756335

[47] Chung C. Y. , Kasten R. W. , and Paff S. M. , et al. *Bartonella* spp. DNA Associated With Biting Flies From California, Emerging Infectious Diseases. (2004) 10, no. 7, 1311–1313, 10.3201/eid1007.030896. 15324557 PMC3323312

[48] Halos L. , Jamal T. , and Maillard R. , et al.Role of Hippoboscidae Flies as Potential Vectors of *Bartonella* spp. Infecting Wild and Domestic Ruminants, Applied and Environmental Microbiology. (2004) 70, no. 10, 6302–6305, 10.1128/AEM.70.10.6302-6305.2004. 15466580 PMC522062

[49] Changbunjong T. , Weluwanarak T. , and Ratanakorn P. , et al.Distribution and Abundance of Stomoxyini Flies (Diptera: Muscidae) in Thailand, The Southeast Asian Journal of Tropical Medicine and Public Health. (2012) 43, no. 6, 1400–1410. 23413703

[50] Changbunjong T. , Sedwisi P. , and Weluwanarak T. , et al.Species Diversity and Abundance of *Tabanus* spp. (Diptera: Tabanidae) in Different Habitats of Thailand, Journal of Asia-Pacific Entomology. (2018) 21, no. 1, 134–139, 10.1016/j.aspen.2017.11.013.

[51] Phasuk J. , Tharawoot T. , and Beaver R. , et al.Seasonal Abundance of Tabanidae (Diptera) on Dairy Farms in Saraburi Province, Thailand, Thai Journal of Agricultural Science. (2011) 44, no. 3, 175–181.

[52] Devendra C. , Smallholder Dairy Production Systems in Developing Countries: Characteristics, Potential and Opportunities for Improvement - Review, Asian-Australasian Journal of Animal Sciences. (2001) 14, no. 1, 104–113, 10.5713/ajas.2001.104.

[53] Gerry A. C. , Predicting and Controlling Stable Flies on California Dairies, 2007, UCANR Publications.

[54] Rochon K. , Hogsette J. , and Kaufman P. , et al.Stable Fly (Diptera: Muscidae)—Biology, Management, and Research Needs, Journal of Integrated Pest Management. (2021) 12, no. 1, 10.1093/jipm/pmab029, 38.

[55] Chakraborty S. , Gao S. , Allan B. F. , Smith R. L. , and Lopez J. E. , Effects of Cattle on Vector-Borne Disease Risk to Humans: A Systematic Review, PLOS Neglected Tropical Diseases. (2023) 17, no. 12, 10.1371/journal.pntd.0011152, e0011152. 38113279 PMC10763968

[56] Sunantaraporn S. , Wacharapluesadee S. , and Maneeorn P. , et al.Molecular Detection and Genetic Diversity of Bat-Associated *Bartonella* spp. in Bat Ectoparasites Collected From Ratchaburi Province, Thailand, Veterinary Medicine and Science. (2026) 12, no. 2, 10.1002/vms3.70815, e70815. 41618733 PMC12859688

