# Supplementary material for: Molecular Detection and Phylogenetic Characterization of Bartonella bovis in Dairy Cattle From Central Thailand
Source: Transbound Emerg Dis. 2026 Aug 6;2026:3831037. doi: 10.1155/tbed/3831037 (PMC13446022; doi:10.1155/tbed/3831037)
Supplement: Supplementary file 1 [file TBED-2026-3831037-s001.docx]

**Supplementary Table S1.** The list of primers used in this study.

| ***Bartonella* genes** | **Primers** | **5’ – 3’** | **Target size (bp.)** | **References** |
| --- | --- | --- | --- | --- |
| *gltA* gene | BhCS.781p | GGGGACCAGCTCATGGTGG | 380 | [1] |
|  | BhCs.1137n | AATGCAAAAAGAACAGTAAACA |  |  |
| *rpoB* gene  (outer reaction) | Btnl_rpoBOF | GTDATGCGTCCAGGTGAGCC | 1,300 | [2] |
|  | Btnl_rpoBOR | CCAAGGCATAAAKGCCACMAGAAC |  |  |
| *rpoB* gene  (inner reaction) | Btnl_rpoBIF | GGTGATAATCTTGGYAATCGWCGCG | 990 |  |
|  | Btnl_rpoBIR | AGCTCACCAACRTGCACRAGAGG |  |  |
| *ribC* gene | BartoribCF | TAACCGATATTGGTTGTGTTGAAG | 585 | [3] |
|  | BartoribCR | TAAAGCTAGAAAGTCTGGCAACATAACG |  |  |

**References**

[1] A. Norman, R. Regnery, P. Jameson, et al., “Differentiation of *Bartonella*-like isolates at the species level by PCR-restriction fragment length polymorphism in the citrate synthase gene,” *Journal of Clinical Microbiology* 33, no. 7 (1995): 1797–1803.

[2] J. Poofery, D. Narapakdeesakul, E. Riana, et al., “Molecular identification and genetic diversity of *Bartonella* spp. in 24 bat species from Thailand,” *Transboundary and Emerging Diseases* 69, no. 4 (2022): e717–e733.

[3] G. Johnson, M. Ayers, S. C. McClure, et al., “Detection and identification of *Bartonella* species pathogenic for humans by PCR amplification targeting the riboflavin synthase gene (*ribC*),” *Journal of Clinical Microbiology* 41, no. 3 (2003): 1069–1072.
